# Items

**Published:** 1900-04

**Authors:** 


### ITEMS.

JUST as the last press work is doing on this edition of the JOURNAL we learn with deep regret, that the firm of D. Appleton Company, the famous publishing house, has been placed in the hands of a receiver. It is gratifying to be assured, however, that the embassassment, chiefly growing out of the plan of selling books on the installment plan, is only temporary. It will be remembered that the house publishes the New York *Medical Journal*, one of our most sterling exchanges, but the *Journal* will not be interrupted in its publication. We certainly hope this great house will soon surmount all its difficulties and continue business with all its old prestige.

Mr. W. Granville Smith, the artist that painted "The Country Doctor," the celebrated picture purchased by the Arlington Chemical Company, of Yonkers, N.Y., makes an interesting announcement in relation to the painting. He has obtained from the Arlington people the necessary permission and rights, and in conjunction with Goupil & Co., of Paris, the most famous art producers in the world, will reproduce the original painting, making the size about 15x25 inches, with ample margin all around, treating the undertaking regardless of expense with a view to obtain the most artistic and elegant result possible. Each copy will be an absolute facsimile of the original, in drawing, color and tonal spirit, on the finest of heavy plate paper, mounted on heavy board, ready for the framer.

The edition will be limited to 250 copies, and each copy will have Mr. Smith's autograph signature. The cost per copy will be $10. Any person desiring to obtain this duplicate, should address Mr. Smith at the studio, Y. M. C. A. Building, 23d St. and 4th Avenue, New York.

---

Messrs. John Wyeth & Bro. desire to call attention to an advertisement that appears in this issue of the Journal relating to Wyeth's prepared food for infants and invalids. They affirm that this food is prepared from malt, milk and cereals, that it is not a predigested food, but one that will digest itself under all conditions. Messrs. Wyeth will be glad to forward a sample and literature to any physicians who would like to give it a practical test.

---

The pen carbon letter book is one of the most useful of the recent inventions offered for the convenience of physicians. With this he may write a letter in the ordinary way and preserve a facsimile copy without waste of time. On page x. of our advertising columns will be found a further reference to the appliance.

---

The Palisade Manufacturing Company, at Yonkers, N. Y., has recently sent out some advertising novelties of interest. Among the group may be mentioned "The Country Doctor," the "Lactopeptine Medical Annual" and the "Essentials of Hematology." The Company especially commends the latter to the profession, as being of great practical value, accurate and modern in its statements, and a guide to blood studies for the general practitioner. This brochure will be furnished by the Company upon application.

---

Mr. James I. Fellows, 48 Vesey Street, New York, well-known as the manufacturer of Fellows's syrup of hypophosphates, has lately issued an attractive brochure, entitled, *The Test of Time and Experience.* It is printed on antique paper with ragged edges and contains much information, relating chiefly to diseases of the respiratory tract in which Fellows's syrup has been found beneficial. It will be sent on application to Mr. Fellows at the above address.

